# DLGAP1 and NMDA receptor‐associated postsynaptic density protein genes influence executive function in attention deficit hyperactivity disorder

**DOI:** 10.1002/brb3.914

**Published:** 2018-01-23

**Authors:** Zili Fan, Ying Qian, Qing Lu, Yufeng Wang, Suhua Chang, Li Yang

**Affiliations:** ^1^ Peking University Sixth Hospital (Institute of Mental Health) National Clinical Research Center for Mental Disorders & Key Laboratory of Mental Health Ministry of Health (Peking University) Beijing China; ^2^ CAS Key Laboratory of Mental Health Institute of Psychology Beijing China; ^3^ Department of Psychology University of Chinese Academy of Sciences Beijing China

**Keywords:** ADHD, cognitive flexibility, *DLGAP1*, executive function, NMDA

## Abstract

**Objective:**

To explore the association of DLGAP1 gene with executive function (EF) in attention deficit hyperactivity disorder (ADHD) children.

**Method:**

A total of 763 ADHD children and 140 healthy controls were enrolled. The difference of EF between ADHD and controls was analyzed using the analysis of covariance (ANCOVA), with IQ, sex, and age as covariates. Both the associations of SNPs with EF and three symptom traits of ADHD were conducted using an additive linear regression model by PLINK with the same covariates as ANCOVA.

**Results:**

Compared with controls, children with ADHD showed poorer cognitive flexibility and inhibition. Two SNPs (rs2049161, *p*‐value = 5.08e‐7, adjusted *p*‐value = 1.63e‐4, rs16946051, *p*‐value = 5.18e‐7, adjusted *p*‐value = 1.66e‐4) survived multiple tests in Trail Making Test. Both SNPs also showed association with TOH (rs2049161, *p* = 6.82e‐4, rs16946051, *p* = 7.91e‐4). Set‐based analysis for gene DLGAP1 and its functional pathway DLGAP1‐DLG4‐NMDA showed they were associated with cognitive flexibility at both gene (*p* = .0057) and pathway level (*p* = .0321). Furthermore, the gene and pathway also showed association with ADHD symptom score. The associated SNPs and their LD proxies were related to the expression of DLGAP1 in medulla and frontal cortex.

**Conclusion:**

Children with ADHD showed deficit in EF, especially, cognitive flexibility and inhibition. DLGAP1 was associated with cognitive flexibility and plan, and the role of DLGAP1 might be implemented through the complex of DLGAP1‐DLG4‐NMDA.

## 1 INTRODUCTION

Attention deficit hyperactivity disorder (ADHD) is one of the most prevalent psychiatric disorders in childhood, which is characterized by hyperactivity, impulsivity, and inattention. The heritability of ADHD is approximately 76% (Faraone & Mick, 2010). Children with ADHD often have executive function (EF) deficits (Barkley, 2010; Willcutt, Doyle, Nigg, Faraone, & Pennington, 2005), including impairment in inhibitory control, working memory, and cognitive flexibility. In recent years, there are many reports about EFs as endophenotype for ADHD (Gau & Shang, 2010). Endophenotype may reduce the heterogeneity of complex neuropsychiatric disorders (Doyle et al., 2008; Rommelse, 2008). A recent meta‐analyses also indicated that some EF impairment was shared across disorders (Snyder, Miyake, & Hankin, 2015). EF refers to a collection of cognitive function. It was a highly heritable trait (Friedman & Miyake, 2017; Friedman et al., 2008). Many genes involved in several neurotransmitter or neuromodulator systems (Darvas & Palmiter, 2015), including glutamatergic, cholinergic (Ragozzino, Mohler, Prior, Palencia, & Rozman, 2009; Wang et al., 2013), serotonergic (Clarke et al., 2005), and dopaminergic signaling (Darvas, Henschen, & Palmiter, 2014; De Steno & Schmauss, 2009), were reported to be associated with EF.

*DLGAP1*, located on chromosome 18p11.31, which is also known as SAP90/PSD‐95‐associated protein (SAPAP) encoding the guanylate kinase‐associated protein (GKAP), was involved in the pathophysiology of several psychiatric disorders, such as obsessive‐compulsive disorder (OCD), schizophrenia (SCZ), major depression disorder (MDD), and Alzheimer's disease (AD; Bertram et al., 2008; Li et al., 2013; Mathias et al., 2016). Although there is no report about the association of DLGAP1 with ADHD, as ADHD shares some genetic basis with other psychiatric disorders, and all these disorders were reported to have impaired cognitive function (Ebmeier, Donaghey, & Steele, 2006; Francazio & Flessner, 2015; Pooragha, Kafi, & Sotodeh, 2013; Thoma, Wiebel, & Daum, 2007), it is valuable to explore the association of *DLGAP1* with ADHD through the underlying impaired cognitive function.

The protein SAPAP1 encoded by *DLGAP1* interacts with PSD95, which is encoded by DLG4 and has been reported to be a predictor of cognitive deficits (Sultana, Banks, & Butterfield, 2009; Whitfield et al., 2014). SAPAPs constitute the member of the N‐methyl‐d aspartate (NMDA) receptor‐associated postsynaptic density proteins. The latter had been showed for potential association with ADHD for its significant role in prefrontal cortex activity and cognitive function (Chang, Lane, & Tsai, 2014; Lehohla, Kellaway, & Russell, 2004). For ADHD model of spontaneously hypertensive rat (SHR), impaired NMDA receptor function in the prefrontal cortex could result in cognitive deficits and an inability to sustaining attention (Lehohla et al., 2004). So the *DLGAP1* may play a role in cognition through DLGAP1‐DLG4‐NMDA complex.

In this study, we firstly compared the difference of several executive function measures among patients with ADHD and controls. Then, we explored the associations between *DLGAP1* variants and performance on the EF relevant measures and further connected with ADHD symptoms to uncover potential genetic mechanisms underlying EF deficit in ADHD individuals. Set‐based analyses for gene and gene‐related pathway were also performed to confirm the association.

## 2 MATERIALS AND METHODS

### 2.1 Participants

All subjects were from the Han Chinese ADHD GWAS project (Yang et al., 2013). Samples were consecutively collected from the child psychiatric outpatient department of Peking University Sixth Hospital. Patients met the diagnostic criteria for ADHD defined by the Diagnostic and Statistical Manual of Mental Disorders‐IV (DSM‐IV). The recruitment procedure of both ADHD cases and controls had been described in our previous publication. In addition, we collected symptom score for the patients with ADHD according to the Clinical Diagnostic Interview Scale (Barkley, 2006) to characterize the severity of the patients. This study was approved by the Institutional Review Board of Peking University Six Hospital. All the parents signed a written informed consent.

### 2.2 Executive function test

As previous literature reported, the children with ADHD showed significant deficits in inhibition, working memory, cognitive flexibility, and plan, and they often were thought to be potential endophenotypes for ADHD (Gau & Shang, 2010; Willcutt et al., 2005). In this study, we conducted digit span test, Trail Making Test (TMT), Stroop test, and Tower of Hanoi on subjects to examine working memory, cognitive flexibility, inhibition, and plan separately.

#### 2.2.1 Trail making test (TMT)

We used TMT to assess cognitive flexibility (Shuai, Chan, & Wang, 2011). The test consisted of two sections (A and B). In section A, the subject was asked to sequentially connect numbers (1–25) randomly scattered on a sheet as quickly as possible. In section B, the subject was asked to connect numbers and letters alternately (i.e., 1–>A–>2–>B–>3–>C, … L–>13; Rane et al., 2016). When the subject made an error, the investigator pointed out immediately before proceeding the test. The times on section A mainly indicated visuoperceptual ability, attention, and motor speed, while that on section B involved set‐shifting. The shifting time was got by the time for section B subtracting that for section A (Anderson, 2001; Sanchez‐Cubillo et al., 2009). Totally, 763 patients with ADHD and 140 normal controls completed TMT.

#### 2.2.2 Tower of Hanoi (TOH)

This task assesses planning and problem‐solving (Bull et al., 2004). Three parallel bars were seated in a board. In the initial position, three disks of different size were put on the left bar with the largest on the bottom and the smallest on the top. The participant was required to move the disk from the left bar to the right bar forming the same tower as beginning. Only one disk could be moved at a time. Bigger disk could not be placed on a smaller one. Total time (TOTIM) to complete the task was recorded. In total, 519 patients with ADHD and 117 normal controls completed TOH.

#### 2.2.3 Stroop test

This task was used to assess inhibition function (Shuai et al., 2011). It included four sessions. Thirty stimuli were presented in a 10 × 3 matrix for three cards each (21 × 29.7 cm). In the first session, the subjects were asked to read the color words (red, green, yellow, and blue) printed in black ink. In the second session, they were asked to name the colored squares (red, green, yellow, and blue). In the third session, the subjects were asked to read the color words printed in different colors. In the fourth session, they were asked to name the colors of the ink. The time required to complete each session was recorded. The color interference time (CIT) equals to the time to complete session 3 minus that for session 1, whereas the word interference time (WIT) equals to the time to complete session 4 minus that for session 2. Totally, 779 patients with ADHD and 140 normal controls completed Stroop Test.

#### 2.2.4 Digit span

This test was used to measure working memory, which was extracted from the Chinese‐Wechsler Intelligence Scale for Children (C‐WISC). The examiner read a series of digit at a rate of one digit per second, and then, the subjects were asked to repeat the digit forward or backward. The length of the digits increased when the participant completed one. The longest digit number achieved was recorded for the forward part and the backward part separately (Trampush, Jacobs, Hurd, Newcorn, & Halperin, 2014). In total, 780 patients with ADHD and 82 normal controls completed digit span test.

### 2.3 Genotyping

Genomic DNA was extracted from peripheral blood using Omega DNA extraction Kit (Omega Bio‐tek Inc., Doraville, GA). Genotyping was performed using the Affymetrix 6.0 array in CapitalBio Inc. (Beijing, China). We extracted the genotype data of SNPs within gene *DLGAP1*. Individuals with call rates <98% or missing IQ, sex, and age information were removed. SNPs with call rate <95%, Hardy–Weinberg equilibrium test *p *<* *.001, or minor allele frequency <1% were removed. Finally, 330 SNPs were remained for association analyses.

### 2.4 Statistics analyses

Comparisons of sex, age, IQ between ADHD and control groups were using chi‐squared or Student's *t* test as appropriately. The differences in executive function measures between ADHDs and controls were analyzed using the analysis of covariance (ANCOVA), with IQ, sex, and age as covariates. As the sample size of controls was much smaller than cases, we also randomly selected the same number of cases with the number of controls for each executive function measures to do the ANCOVA to confirm the result. Principal component analysis (PCA) for the executive function measures was performed in SPSS version 19 (http://www-01.ibm.com/software/uk/analytics/spss/, RRID: SCR_002865). All measures were normalized before the PCA. Association analysis of *DLGAP1* SNPs with cognitive measures and ADHD symptom traits were conducted using an additive linear regression model by PLINK (Purcell et al., 2007) version 1.0.7 (http://pngu.mgh.harvard.edu/~purcell/plink/, RRID: SCR_001757), with the above covariates. The *p*‐value of SNP was adjusted by Bonferroni correction by multiplying the number of SNPs we tested. To present the regional plot, the nongenotyped SNPs within *DLGAP1* were imputed using MACH‐admix 1.0 (http://www.unc.edu/~yunmli/MaCH-Admix/, RRID:SCR_009598; Liu, Li, Wang, & Li, 2013) using the ASN data (286 individuals) from the 1000 Genomes Project Integrated Phase 1 Release 37 as the reference panel. Imputed SNPs with a squared correlation between imputed and true genotypes (rsq) <0.6 or SNPs with minor allele frequency <0.01 were removed. Set‐based association analysis for *DLGAP1* was conducted for all 330 SNPs within gene *DLGAP1* using the −set‐test in PLINK. Default parameters for the max #SNPs, *r*‐squared, and *p*‐value were used (−set‐max 5, −set‐*r* ^2^ .5, −set‐*p* .05). Nine genes (*DLGAP1, DLG4, GRIN1, GRIN2A, GRIN2B, GRIN2C, GRIN2D, GRIN3A,* and *GRIN3B*) were included in pathway DLGAP1‐DLG4‐NMDA (Stephenson, 2006). Totally 700 SNPs within these nine genes were used for the set‐based analysis of this pathway using the −set‐test in PLINK. The max #SNPs was set to 30; other parameters were as default.

### 2.5 Functional analysis

To explore the function of the significant SNP on the expression of *DLGAP1*, we got the SNPs in linkage disequilibrium (LD) with the significant SNP (*r* ^2^ *>* .8) using the 1000 Genomes Project ASN population data (http://www.1000genomes.org/, RRID: SCR_006828; Genomes Project et al., 2012); then, we searched these SNPs in the UK Brain Expression Cohort (UKBEC) data set (GSE46706; Trabzuni et al., 2011). Detailed processing and exclusion criteria were described elsewhere (Trabzuni et al., 2011). eQTL analysis was described by Ramasamy et al. (2014). The eQTL data and expression plot were obtained from BRAINEAC (http://www.braineac.org/) by searching *DLGAP1*, selecting its transcript, and then stratifying expression by the significant SNP or its LD proxy. The gene expression profile of *DLGAP1* was searched in GTEx (http://www.ncbi.nlm.nih.gov/gtex/GTEX2/gtex.cgi, RRID: SCR_001618; Consortium, 2015) and BRAINEAC.

## 3 RESULTS

### 3.1 Executive function feature in ADHD children

We collected the Tb‐Ta time in TMT for 763 patients with ADHD and 140 normal controls. The comparison of demographic characteristics and performance on the TMT between ADHD and control groups are displayed in Table 1. There was no difference found except that the proportion of males in ADHD group appeared to be larger than control group (*p *<* *.001), and the average IQ of the control group was higher than that of the ADHD group (*p *<* *.005). After controlling for age, IQ, and gender, performance of the TMT test showed significant difference between ADHD and control groups (*p *=* *.009). By randomly selecting 140 of 763 cases, the performance difference of TMT test still existed between the 140 cases and 140 controls. Furthermore, we collected another five executive function task measures for patients with ADHD, including total time in TOH, word interference time (WIT), color interference time (CIT), digit span forward (DSDF), and digit span backward (DSDB). The sample demographic information and the comparison of these EF measures between case and control groups are shown in Table 1. The Stroop performances were significantly different between patients with ADHD and controls. After normalization for these six measures, principle component analysis (PCA) was conducted. Two principle components were detected. The component plot in the rotated space and rotated component matrix is shown in Figure S1. The first principle component was mainly related to Tb‐Ta time and TOH time, while the second principle component was mainly related to CIT and WIT. DSDB and DSDF could not be differentiated using these two PCs.

**Table 1:** Comparison of characteristics and performance on executive function tests between ADHD and control groups

	ADHD	Controls	χ^2^/*t*/*F*	*p*
Sex (male:female)	671:114	79:61	65.36	<.001
Age (Month) (mean [*SD*])	118.46 ± 28.92	116.88 ± 21.23	0.618	0.537
IQ (mean [*SD*])	104.36 ± 14.61	117.22 ± 13.60	9.693	<.001
Shifting time	147.09 ± 108.05	90.53 ± 83.92	6.94	.009
Total time for TOH	183.75 ± 130.86	164.54 ± 120.00	0.076	.78
CIT	7.00 ± 9.75	3.97 ± 5.20	8.96	.003
WIT	30.25 ± 17.50	20.95 ± 9.24	27.06	<.001
DSDF	7.75 ± 1.42	7.8 ± 1.50	0.94	.333
DSDB	4.32 ± 1.53	4.68 ± 1.30	0.93	.364

TOH, Tower of Hanoi; CIT, color interference time in Stroop; WIT, word interference time in Stroop; DSDF, digit span forward; DSDB, digit span backward. Sample size for shifting time: *N*_ADHD_ = 763, *N*_control_ = 140; TOH: *N*_ADHD_ = 519, *N*_control_ = 117; Stroop test: *N*_ADHD_ = 779, *N*_control_ = 140; digit span: *N*_ADHD_ = 780, *N*_control_ = 82.

### 3.2 Association between DLGAP1 gene and components of executive function

**Figure 1:**
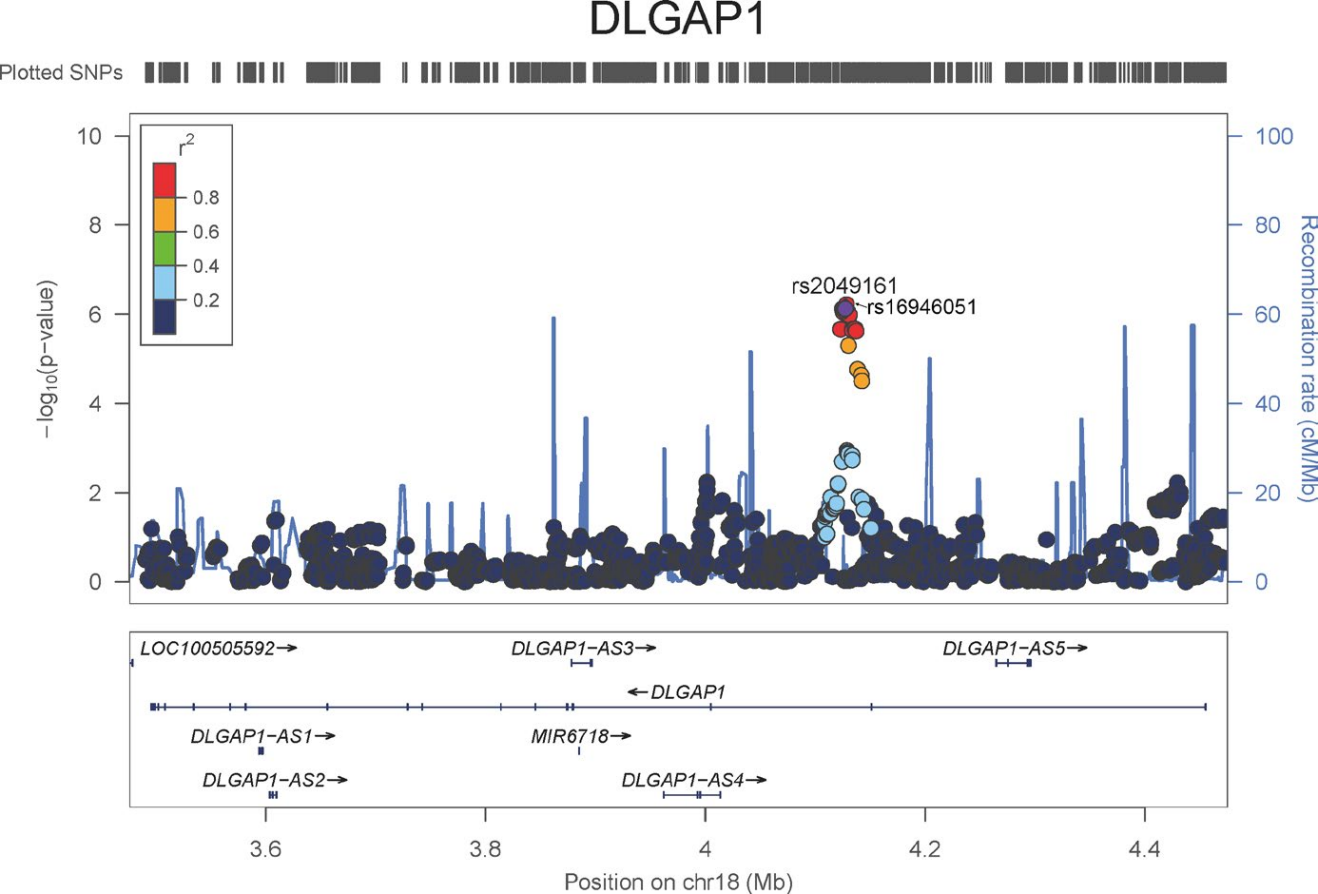
Regional plot for SNP rs2049161, rs16946051 in *DLGAP1*. The SNPs after imputation were also plotted

As the small sample size of controls, the association analysis was performed only in cases. Association analysis for the 330 SNPs within gene *DLGAP1* with the shifting time in 763 ADHD children identified two SNPs (rs2049161, *p *=* *5.08e‐7, adjusted *p *=* *1.63e‐4, rs16946051, *p *=* *5.18e‐7, adjusted *p *=* *1.66e‐4) to be significant after Bonferroni multiple testing correction (Table 2, the regional plot is shown in Figure 1). These two SNPs were in high LD (*r* ^2^ > .8). Association analyses of the above two SNPs with another five executive function measures showed these two SNPs were associated with the TOH total time (*p *=* *6.81e‐4 for rs2049161, *p *=* *7.91e‐4 for rs16946051, Table 2), but not for WIT, CIT, DSDB, and DSDF. Furthermore, we checked the association of the *DLGAP1* SNPs with the two principle components of the six normalized executive function measures. The result showed the two significant SNPs for cognitive flexibility were significantly associated with the first principle component (*p *=* *6.681e‐05 for rs2049161, *p *=* *5.1e‐05 for rs16946051), but not the second principle component.

**Table 2:** Association result for SNP rs2049161 and rs16946051 with shifting time in TMT and total time for TOH

SNP	Position (hg19)	A1	EF measures	Phenotype mean (*SD*)	Frq	BETA	L95	U95	*p*
rs2049161	4127583	G	Shifting time in TMT	GG: 205 (184)	0.1189	38.83	23.81	53.85	5.08E‐07
				GT: 172 (137)					
				TT: 139 (95)					
			Total time for TOH	GG: 295 (280)	0.1196	40.89	17.44	64.34	6.81E‐04
				GT: 204 (136)					
				TT: 176 (124)					
rs16946051	4128633	A	Shifting time in TMT	AA: 197 (176)	0.1166	38.58	23.65	53.52	5.18E‐07
				AG: 173 (137)					
				GG: 139 (95)					
			Total time for TOH	AA: 275 (269)	0.1175	40.07	16.81	63.33	7.91E‐04
				AG: 206 (137)					
				GG: 176 (124)					

Freq is the allele frequency of the SNP in those samples with TMT or TOH result. L95 is the lower boundary of 95% confidence interval of BETA; U95 is the upper boundary of 95% confidence interval of BETA.

### 3.3 Set‐based analysis for DLGAP1 and pathway DLGAP1‐DLG4‐NMDA

As the DLGAP1 SNPs were mainly associated with cognitive flexibility among all EF components, we further did set‐based analysis for gene DLGAP1 and complex DLGAP1‐DLG4‐NMDA for cognitive flexibility. Set‐based association analysis for all SNPs in *DLGAP1* showed the *p*‐value .0057. Nine genes (*DLGAP1, DLG4, GRIN1, GRIN2A, GRIN2B, GRIN2C, GRIN2D, GRIN3A,* and *GRIN3B*) were used for the set‐based association analysis of the complex DLGAP1‐DLG4‐NMDA. The resulted *p*‐value of .0321 indicated the effect of this pathway in the variability of cognitive flexibility. At the same time, Set‐based association analysis for all SNPs in *DLGAP1* and pathway level showed the *p*‐value .005 and .0079, respectively, with principle component analysis components, which further demonstrated the association of this gene with EF.

### 3.4 Association of DLGAP1 with ADHD symptoms

To test whether the *DLGAP1* SNPs associated with cognitive flexibility were also associated with ADHD and ADHD symptom, we extracted the *p*‐values of these two significant SNPs in PGC ADHD data set and our Han Chinese ADHD data set (Yang et al., 2013). We also tested the association of them with ADHD symptom total score. All these SNP level results were not significant (*p *>* *.05). The set‐based analysis result showed significance for the association of gene *DLGAP1* with the total score of ADHD symptom (*p *=* *.0481). When we enlarged the parameters of set‐based analysis (set −sex‐max 50, −set‐*r* ^2^ .3 and −set‐*p* .1) to include more SNPs, the set‐based analysis for the pathway DLGAP1‐DLG4‐NMDA also showed significance in ADHD symptom score (*p *=* *.0313).

### 3.5 Function of the variants and gene

We searched the eQTL data for the two significant SNPs to explore whether they regulated the expression of *DLGAP1*. We found the different alleles of these two SNPs had significant effect on the expression of *DLGAP1* in medulla (Figure S2). eQTL data for all SNPs with high LD (*r* ^2^ > .8) with the significant SNP rs2049161 showed these SNPs were also correlated to the expression of *DLGAP1* in frontal cortex (Table S1). In normal samples, *DLGAP1* gene was mainly expressed in brain. The highest expression level was found in frontal cortex (Figure S3a). We further checked the expression of *DLGAP1* in different brain region in BRAINEAC. The regions with the highest expression level included temporal cortex, frontal cortex, and occipital cortex (Figure S3b). The variant we identified may regulate the expression of *DLGAP1* and further contribute to cognitive flexibility dysfunction.

## 4 DISCUSSION

In this study, we firstly detected the deficit of cognitive flexibility and inhibition in our ADHD sample as expected, which had been consistently reported in previous studies (Paloscia et al., 2013). But we did not found this difference in our sample in working memory and plan, which might be due to our small sample size, especially small control sample size.

Then, we explored the association between *DLGAP1* and EF in children with ADHD. We identified two significant SNPs of *DLGAP1* with cognitive flexibility. The association was also observed for the time to complete the TOH. The TOH mainly evaluate planning, which is one of a high‐order EF, including the component of cognitive flexibility (Diamond, 2013). During the test of TOH, participants should shift between subgoals to achieve the final goal in the test. Bishop, Aamodtleeper, Creswell, Mcgurk, & Skuse (2001) indicated that shifting between different subgoals might be a better predictor of TOH performance. Bull, Espy, & Senn (2004) further demonstrated cognitive flexibility among mental sets was associated with TOH performance. So, the association results in TOH probably support that in cognitive flexibility. We did not find the association of DLGAP1 with inhibition. Inhibition is the most fundamental component of EF. Cognitive flexibility was based on inhibition, but it showed diversity component (Friedman & Miyake, 2017). So the components of EF might have different genetic contributors. Our results provided evidence that the diversity components might have different genetic influences, and different sets of genes might contribute to the various EF, which further distinguish cognitive function. This is coincidence within first twin study of latent EF variables (Friedman et al., 2008).

We further checked the association of *DLGAP1* and pathway DLGAP1‐DLG4‐NMDA with ADHD symptom. The result showed significant association of them with ADHD symptom score. The ADHD symptom was related to inhibition, shifting, and emotional regulation (Chhabildas, Pennington, & Willcutt, 2001; Riccio, Homack, Jarratt, & Wolfe, 2006; Toplak et al., 2009). The association of *DLGAP1* with ADHD symptom might be mediated by its effect on cognitive function.

The expression data for *DLGAP1* support its function in brain. The two SNPs found significantly associated with cognitive flexibility had significant effect on the expression of *DLGAP1* in medulla. A noradrenaline projection from medulla to nucleus accumbens was reported to play a role in stress response and behavioral flexibility (Stahl). Besides these two SNPs, those in LD with them were found to regulate the expression of *DLGAP1* in frontal cortex, which is consistent with the report that genes involved in synaptic signaling or plasticity in prefrontal cortex (PFC) had been implicated in cognition (Logue & Gould, 2013). And in the recent review about DLGAP family, it also summed up that DLGAP1 is thought to be involved in SCZ starting from nucleus accumbens, AD in frontal cortex, and MDD originating from hippocampus (Rasmussen, Rasmussen, & Silahtaroglu, 2017).

As *DLGAP1* encoding protein SAPAP interacted with PSD95 encoded by *DLG4*, that constituted NMDA receptor‐associated postsynaptic density proteins, *DLG4* was reported to be a predictor of cognitive deficits (Sultana et al., 2009; Whitfield et al., 2014), while NMDA was suggested for the association with ADHD and prefrontal dysfunction, we tested the association of DLGAP1‐DLG4‐NMDA pathway with cognitive flexibility and ADHD symptom. The results validated our hypothesis. NMDA dysfunction has been considered to be involved in ADHD pathogenesis for its effect on neurodevelopment, attentional circuitry, and impulse inhibition (Chang et al., 2014). Besides, NMDA alteration (subunit expression, activity, and localization) in the PFC had also been implicated in many neuropsychiatric disorders with impairment of cognitive flexibility, including SCZ, depression, and anxiety disorders (Balu & Coyle, 2014; Belforte et al., 2010; Beneyto, Kristiansen, Oniorisan, Mccullumsmith, & Meadorwoodruff, 2007; Bitanihirwe, Lim, & Woo, 2010; Davis, 2011; Laruelle, 2014). Decreased PSD95 expression in prefrontal cortex was also found in SCZ and MDD (Feyissa, Chandran, Stockmeier, & Karolewicz, 2009; Ohnuma et al., 2000). These data further provide evidence for possible involvement of DLGAP1‐DLG4‐NMDA in cognitive flexibility and ADHD. (Lee et al., 2013). In conclusion, our study confirmed children with ADHD have cognitive flexibility and inhibition deficits contrary to the healthy control, which is associated with *DLGAP1* gene. The significant SNPs and those in LD with them influenced *DLGAP1* expression in wide brain regions including medulla and frontal cortex. The role of *DLGAP1* seems to be modulated by the complex of DLGAPs‐DLG4‐NMDA to contribute to cognitive flexibility and ADHD symptom, which may provide new target for the treatment of ADHD. Further investigation into the cognitive phenotype may provide new pathway to the discovery of ADHD genes.

## CONFLICT OF INTEREST

The authors declare no conflict of interests.
