# Supplementary material for: DLGAP1 and NMDA receptor‐associated postsynaptic density protein genes influence executive function in attention deficit hyperactivity disorder
Source: Brain Behav. 2018 Jan 23;8(2):e00914. doi: 10.1002/brb3.914 (PMC5822579; doi:10.1002/brb3.914)
Supplement: Supplementary file 1 [file BRB3-8-e00914-s001.doc]

**Supplementary Information**

***DLGAP1* and NMDA receptor – associated postsynaptic density protein genes influence executive function in attention deficit hyperactivity disorder**

Zili Fan^1,#^, Ying Qian^1,#^, Qing Lu^1^, Yufeng Wang^1^, Suhua Chang^2,3,*^, Li Yang^1,*^

^1^Peking University Sixth Hospital (Institute of Mental Health), National Clinical Research Center for Mental Disorders & Key Laboratory of Mental Health, Ministry of Health (Peking University), 51 HuayuanBei Road, Beijing 100191, China.

^2^CAS Key Laboratory of Mental Health, Institute of Psychology, 16 Lincui Road, Beijing 100101, China.

^3^Department of Psychology, University of Chinese Academy of Sciences, 19 A Yuquan Road, Beijing 100049, China.

#: These authors contributed equally.

*: Correspondence authors: Li Yang, 51 HuayuanBei Road, Beijing 100191, China, email: [yangli_pkuimh@bjmu.edu.cn](mailto:yangli_pkuimh@bjmu.edu.cn); Suhua Chang, 16 Lincui Road, Beijing 100101, China, email: [changsh@psych.ac.cn](mailto:changsh@psych.ac.cn)

**Figure** S1 The component plot in the rotated space and rotated component matrix for the normalized scores for shift, TOTIM, CIT, WIT, DSDB and DSDF. The principle component (PCA) was conducted using SPSS. The phenotype values of the traits were normalized before the PCA.


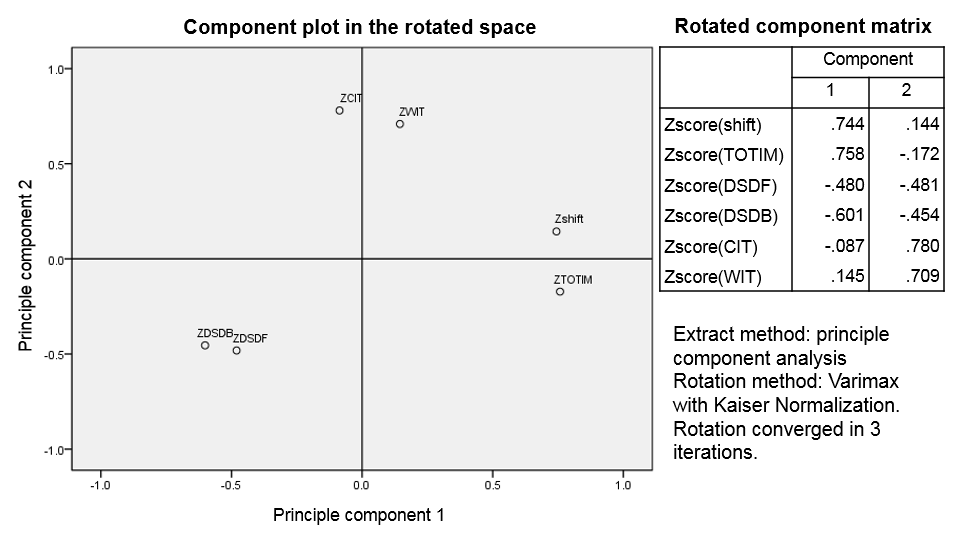


**Figure S2** eQTL plot for the two significant loci (rs2049161 and rs16946051) in brain. TCTX: temporal cortex, FCTX: frontal cortex, OCTX: occipital cortex, CRBL: cerebellar cortex, HIPP: hippocampus, PUTM: putamen, THAL: thalamus, MEDU: medulla, SNIG: substantianigra, WHMT: intralobular white matter.

**
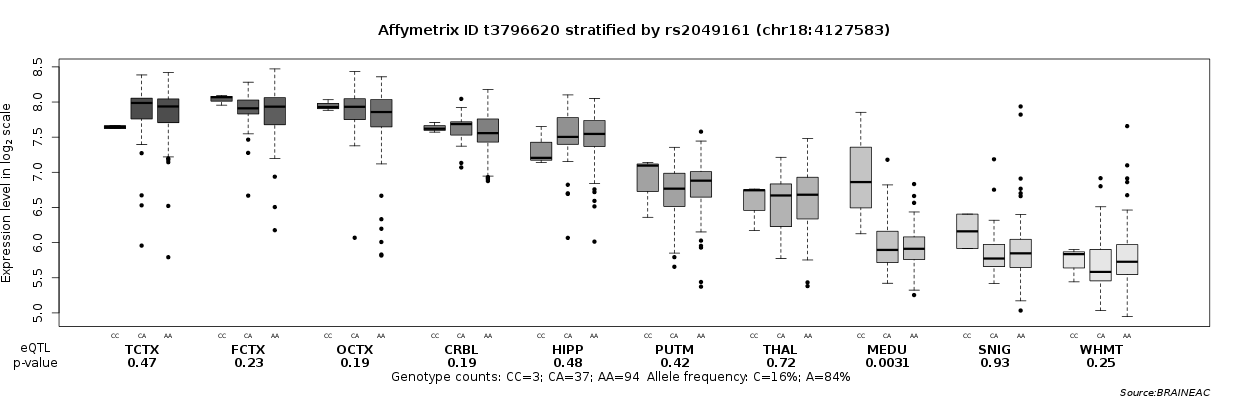
**


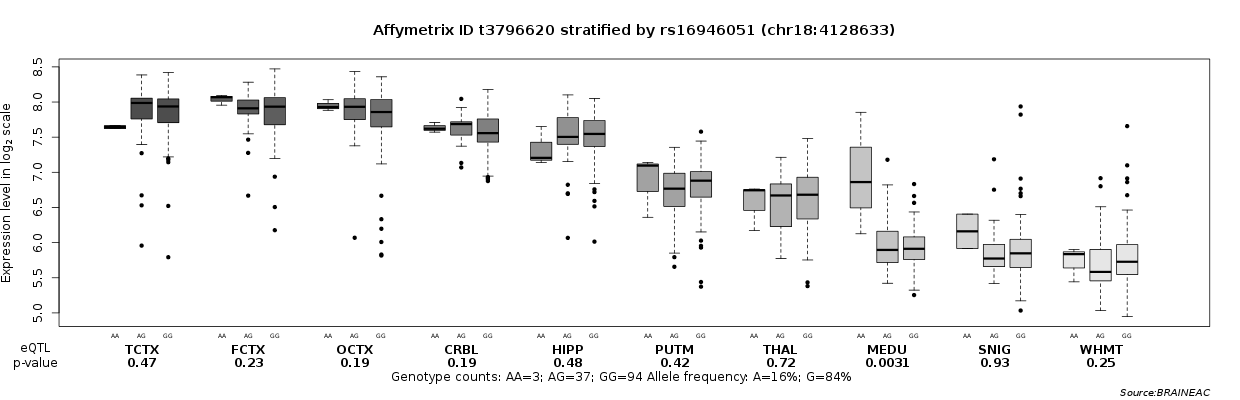


**Figure S3** *DLGAP1* gene expression level in different tissues of human (A) and in different brain regions. A was plotted using GTEx, B was generated by using the BRAINEAC (<http://www.braineac.org/>).

**
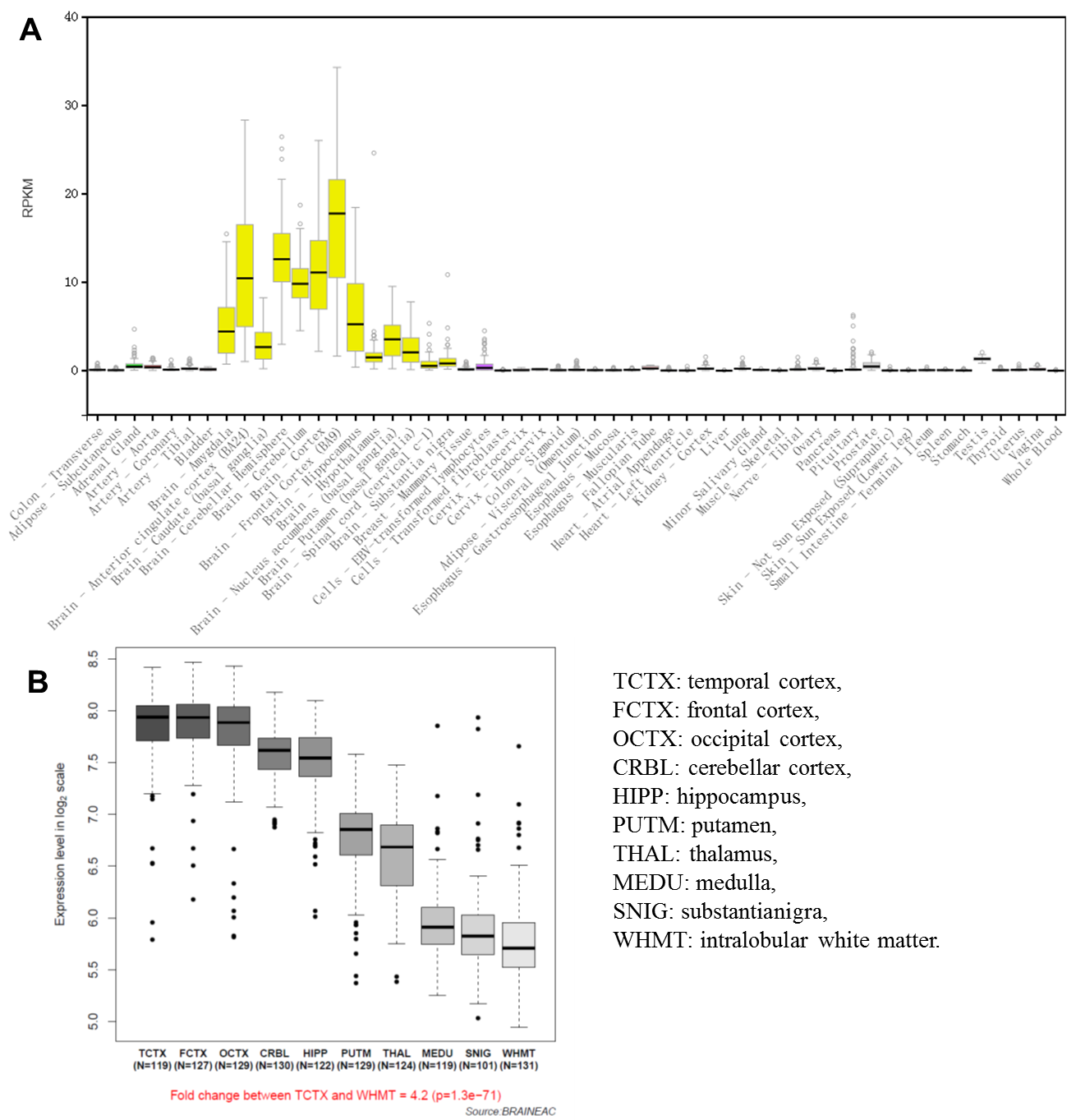
**

**Table S1** eQTL information for SNPs with high LD (*r^2^*>8) with the the significant SNP rs2049161. Data was extracted from BRAINEAC.

| **Tissue** | **SNP** | **Gene** | **Trans/cis** | ***P*-value** |
| --- | --- | --- | --- | --- |
| frontal cortex | rs1116153 | DLGAP1 | cis | 0.041 |
| medulla | rs1116153 | DLGAP1 | cis | 0.0037 |
| medulla | rs1607965 | DLGAP1 | cis | 0.003 |
| medulla | rs16946051 | DLGAP1 | cis | 0.0031 |
| medulla | rs2049161 | DLGAP1 | cis | 0.0031 |
| medulla | rs73386719 | DLGAP1 | cis | 0.0031 |
| medulla | rs8087631 | DLGAP1 | cis | 0.0031 |
| frontal cortex | rs9957929 | DLGAP1 | cis | 0.045 |
| medulla | rs9957929 | DLGAP1 | cis | 0.0055 |
| frontal cortex | rs9958677 | DLGAP1 | cis | 0.041 |
| medulla | rs9958677 | DLGAP1 | cis | 0.0039 |
| medulla | rs35295677 | DLGAP1 | cis | 0.0032 |
